# M1 macrophages evoke an increase in polymeric immunoglobulin receptor (PIGR) expression in MDA-MB468 breast cancer cells through secretion of interleukin-1β

**DOI:** 10.1038/s41598-022-20811-6

**Published:** 2022-10-07

**Authors:** Wichitra Asanprakit, Dileep N. Lobo, Oleg Eremin, Andrew J. Bennett

**Affiliations:** 1 FRAME Alternatives Laboratory, Faculty of Medicine and Health Sciences, School of Life Sciences, University of Nottingham, Nottingham, UK; 2 Nottingham Digestive Diseases Centre and National Institute for Health Research (NIHR) Nottingham Biomedical Research Centre, Nottingham University Hospitals NHS Trust and University of Nottingham, E Floor, West Block, Queen’s Medical Centre, Nottingham, UK; 3 Department of Surgery, Phramongkutklao Hospital and College of Medicine, Bangkok, Thailand; 4 MRC Versus Arthritis Centre for Musculoskeletal Ageing Research, School of Life Sciences, University of Nottingham, Queen’s Medical Centre, Nottingham, UK

**Keywords:** Cancer, Immunology, Medical research

## Abstract

High expression of polymeric immunoglobulin receptor (PIGR) in breast cancer is associated with increased 5-year survival rate. However, the factors influencing PIGR expression in breast cancer have not been elucidated. The aim of this study was to determine the role of macrophages and cytokines affecting expression of PIGR in two breast cancer cell lines. M1, M2 macrophage conditioned media (CM) and recombinant human cytokines were used to determine factors which increased PIGR expression in MCF7 (HTB-22) and MDA-MB468 (HTB-132) breast cancer cell lines. The level of PIGR expression in the cells and PIGR secretory component were evaluated by real-time quantitative polymerase chain reaction and Western blotting. M1 macrophage CM induced a dose-dependent increase in PIGR mRNA expression in MDA-MB468 cells, up to 20-fold. The level of PIGR expression in MCF7 cells was very low and not affected by M1 and M2 CM. Interferon gamma (IFN-γ) and interleukin (IL)-1β also increased PIGR expression in MDA-MB468 and MCF7 cells. However, IL-1β was demonstrated to increase in M1 macrophages, while IFN-γ was not. The role of IL-1β secreted from M1 macrophages in increasing expression of PIGR was confirmed by IL-1 receptor blockade, indicating that IL-1β was the major M1 macrophage-derived cytokine that enhanced PIGR expression. Elevated PIGR expression in breast cancer in vivo may reflect the polarization state of tumor-associated immune cells.

## Introduction

The polymeric immunoglobulin receptor (PIGR) is a transmembrane protein, which is expressed on the surface of glandular epithelial cells^1^. It modulates transcytosis of polymeric immunoglobulin (pIg) molecules (IgA and to a lesser extent IgM) produced from plasma cells in the lamina propria^2^. PIGR binds to pIgA at the basolateral surface of epithelial cells and then endocytoses and transcytoses to present on the apical surface. At the apical surface, the extracellular ligand binding portion of PIGR is cleaved by proteolytic enzymes and released as a secretory component (SC) in free-form or as part of secretory IgA (SIgA) to the lumen^2^. SIgA acts as the first line of antigen specific immunological defense to protect the mucosal surface against pathogens, toxic substances, and antigens that could harm the human body^3^. Free SC protects SIgA from proteolytic degradation and also has innate anti-microbial properties. PIGR, therefore, plays a major role in the mucosal immune system and links innate and adaptive immunity^2^.

The alteration of PIGR expression, either increased or decreased, has been shown in various malignant and premalignant lesions and is related to cancer outcomes. Elevated PIGR expression in hepatocellular cancer, colon cancer, osteosarcoma and glioma was demonstrated to correlate with poor prognosis^4–7^. In contrast, many studies have reported favorable outcomes associated with increased PIGR expression in patients with upper gastrointestinal tract, pancreatic, periampullary, lung, endometrial and ovarian cancers^8–14^.

Breast cancer was one of the earliest cancers shown to express PIGR. Both invasive breast cancer cells and metastatic cancer cells in axillary lymph nodes have demonstrated intense immunofluorescence staining of SC^15^. Plasma concentration of SC in patients with metastatic breast cancer was found to be higher than in healthy women and related to the clinical course of the disease^16^. A study showed that high expression of PIGR mRNA was a favorable prognostic factor in patients with breast cancer^17^. There is, however, no documentation regarding the regulatory factors that influence PIGR expression in breast cancer.

Macrophages are the major component of immune infiltrating cells in the tumor microenvironment (TME), comprising up to 50% of the tumor mass^18^. Macrophage phenotypes and functions encompass a wide-ranging spectrum from proinflammatory macrophages, which have microbicidal and tumoricidal capacity (M1 or classically activated macrophages) to anti-inflammatory macrophages, which have immunosuppressive and tumor promoting effects (M2 or alternatively activated macrophages)^19,20^. Lipopolysaccharide (LPS) and/or interferon gamma (IFN-γ) can polarize macrophages to be M1 macrophages, while interleukin (IL)-4 and/or IL-13 are responsible for M2 polarization^21,22^. The functional heterogeneity of macrophages is dynamic and depends on differential regulation by environmental factors. On the other hand, macrophages themselves have a broad range of effects on the TME and tumor cells. In the TME, M1 macrophages can release lytic factors which cause tumor cell lysis directly and also secrete factors/cytokines which indirectly activate anti-tumor effects of other cell types^23^. Infiltration of CD11c+ tumor associated macrophages (TAMs) (M1 macrophages) in the tumour stroma of invasive breast cancer led to a good prognosis as regards overall survival and disease free survival^24^. In large and locally advanced breast cancer, high number of suppressor of cytokine signaling 3+ (SOCS3+) TAMs (M1 macrophages) were associated with a pathological complete response after neoadjuvant chemotherapy^25^.

PIGR overexpression in breast cancer was shown to be related with favorable outcome. The upregulation of PIGR is influenced by surrounding inflammatory stimuli. The proinflammatory cytokines including IFN-γ, tumor necrosis factor-α (TNF-α), IL-1β have been shown to be key cytokines in PIGR regulation in intestinal epithelial cells^2,26^. Our hypothesis was that increasing of M1 macrophages, which produce proinflammatory cytokines in the TME, will reflect in increased PIGR expression in breast cancer cells which may be a surrogate biomarker of a favorable outcome in patients with breast cancer. The aim of this study was to determine the role of macrophages and cytokines affecting expression of PIGR in two breast cancer cell lines.

## Methods

### Cell lines

MCF7 (HTB-22) and MDA-MB468 (HTB-132) breast cancer cell lines were purchased from the American Type Culture Collection (ATCC, Manassas, VA, USA). Murine bone marrow derived macrophages (BMDMs) were kindly provided by Dr Luisa Martinez-Pomares, Faculty of Medicine and Health Sciences, University of Nottingham. MCF7 cells were cultured in Eagle’s minimum essential medium (EMEM) (M2279; Sigma-Aldrich, St Louis, MO, USA) supplemented with 10% fetal bovine serum (FBS), 0.01 mg/ml human recombinant insulin, 2 mM l-glutamine, 1 mM sodium pyruvate, 1% minimum essential medium non-essential amino acids. MDA-MB468 cells were cultured in Dulbecco’s modified Eagle medium (DMEM) (D6046; Sigma-Aldrich) supplemented with 10% FBS. BMDMs were cultured in Roswell Park Memorial Institute medium (RPMI) 1640 (R0883; Sigma-Aldrich) supplemented with 10% FBS, 2 mM l-glutamine, 100 U/ml penicillin and 100 µg/ml streptomycin sulphate. All cell lines were incubated in a humidified atmosphere containing 5% CO_2_ at 37 °C.

### Macrophage polarization

BMDMs were plated at a density of 8 × 10^4^ cells/well in 12-well plates and incubated for 5 h. Cell culture media were changed to fresh media containing LPS 50 ng/ml (Sigma-Aldrich) or IL-4 20 ng/ml (Peprotech, Rocky Hill, NJ, USA), in order to polarize BMDMs toward M1 or M2 subtypes, respectively. To demonstrate the efficiency of the polarization, IL-1β, inducible nitric oxide synthase (iNOS), IL-10 and Arginase 1 (ARG1) mRNA expression in BMDMs after treatment with LPS or IL-4 were evaluated by Taqman real-time quantitative polymerase chain reaction (RT-qPCR). Untreated BMDMs served as the control. IL-1β and iNOS are markers for M1 polarization, while IL-10 and ARG1 are markers for M2 polarization^22,27,28^.

### Preparation of macrophage-conditioned medium

BMDMs were plated in T175 flasks at a cell density of 4 × 10^6^ cells/flask in 35 ml medium and incubated for 5 h. M1 and M2 polarized macrophages were gently washed with warm phosphate buffer saline (PBS) thrice. 35 ml of fresh medium were added and conditioned medium (CM) was collected after 24 h of incubation. CM was passed through a 0.22 µm syringe filter and stored at − 80 °C until used. CM prepared from untreated/non-polarized BMDMs was designated M0 CM.

CM was used at 25, 50 and 100% concentrations as culture medium for MCF7 and MDA-MB468 cells for 48 h. Additionally, cells were treated with recombinant human IFN-γ 10 ng/ml, IL-1β 10 ng/ml, tumor necrosis factor alpha (TNF-α) 10 ng/ml, IL-10 20 ng/ml (all Peprotech) or transforming growth factor beta (TGF-β) 5 ng/ml (R&D, Minneapolis, MN, USA). Cells were incubated for 24 or 48 h before being harvested.

### IL-1 receptor blockade assay

MCF7 and MDA-MB468 cells were plated in 12-well plates at a cell density of 2 × 10^5^ cells/well in 1 ml medium and incubated overnight. The cells were treated first with recombinant human IL-1 receptor antagonist (IL-1RA) (Peprotech) 500 ng/ml for 30 min and then with M1 CM together with IL-1RA 500 ng/ml. These were compared with treatment with M1 CM alone, with no IL-1RA. The cells were incubated for 48 h before being harvested for further assays.

### RNA isolation and cDNA synthesis via reverse transcription

Total RNA was isolated from cells using TRI Reagent (Sigma-Aldrich) according to the manufacturer’s instructions. 500 ng of total RNA was reverse transcribed to first-strand cDNA using Affinity-Script Multiple Temperature cDNA Synthesis Kit (Agilent Technologies, Santa Clara, CA, USA) according to the manufacturer’s instructions.

### Taqman real-time quantitative polymerase chain reaction

Taqman RT-qPCR was performed to measure and quantify gene expression. Primers and probe sequences were designed using Primer Express Software Version 3.0.1 (Applied Biosystems Inc., Foster City, CA, USA https://www.thermofisher.com/uk/en/home/technical-resources/software-downloads/primer-express-software-download.html) (Supplementary Table 1). Precision FAST qPCR Master Mix (Primerdesign, Camberley, UK) and AriaMx Real-Time PCR machine (Agilent Technologies) were used to performed RT-qPCR. Each reaction included 3 µl of cDNA, 6.5 µl of master mix, 0.4 µl of forward primer (10 µM), 0.4 µl of reverse primer (10 µM), 0.25 µl of probe (10 µM) and 2.45 RNase-free water. The RT-qPCR conditions comprised a preliminary cycle of 95 °C for 2 min followed by 40 cycles of 95 °C for 5 s and 60 °C for 20 s. The target gene expression was normalized with human GAPDH or murine β-actin and gene expression was calculated using the relative standard curve method^29^.

### Protein isolation from cell lysates

Cells were washed thrice with ice cold PBS and lysed with radioimmunoprecipitation assay (RIPA) buffer [10 mM Tris-HCl, 150 mM NaCl, 0.5% Na deoxycholate, 1% Triton X-100, 0.1% sodium dodecyl sulfate (SDS) and 1× protease inhibitor]. Cell lysate was sonicated thrice on ice for 10 s and incubated at 4 °C using an end over end rotator for 45 min. Centrifugation was performed at 21,000×*g* for 15 min at 4 °C to pellet the debris. The supernatant was collected for further analysis and stored at − 20 °C. Protein was quantified using a Pierce BCA Protein Assay Kit (Thermo Fisher Scientific, Waltham, MA, USA) according to the manufacturer’s instructions.

### Concentration of protein in cell culture media

In order to concentrate proteins for Western blotting analysis, MCF7 and MDA-MB468 cells were cultured as previously described. After treatment with cytokine for 48 h, 5 ml of cell culture media was collected and centrifuged at 5000 rpm for 5 min to remove cell debris. Supernatant was collected and concentrated using Vivaspin 2, 10,000 molecular weight cut off (MWCO), polyethersulfone (PES) membrane (Sartorius, Göttingen, Germany) by centrifugation in a swing bucket at 3901×*g* at 20 °C until the concentrated sample was less than 45 µl.

### Western blotting

Protein samples were separated on a 10% SDS-polyacrylamide gel and then transferred to nitrocellulose membrane (GE Healthcare, Chicago, IL, USA). Membrane was blocked with 5% skimmed milk in Tris-buffered saline with Tween (TBST) (25 mM Tris, 200 mM NaCl, 2.7 mM KCl, 0.1% Tween 20) for 1 h at room temperature before incubation with primary antibody (goat anti-human PIGR antibody [R&D Systems 1:5000] or mouse anti-human α-tubulin antibody [Sigma-Aldrich 1:500]) overnight at 4 °C. After washing three times with TBST, the membrane was incubated with horseradish peroxidase (HRP) conjugated secondary antibody (rabbit anti-goat IgG HRP-conjugated antibody [R&D Systems 1:1000] or goat anti-mouse IgG HRP-conjugated antibody [Sigma-Aldrich 1:70,000]) for 1 h at room temperature. The membrane was washed thrice with TBST and enhanced chemiluminescent detection was performed using Immobilon Western chemiluminescent HRP substrate (Millipore, Burlington, MA, USA) according to the manufacturer’s instructions. The chemiluminescent signal was visualized using a Luminescent Image Analyzer (Fujifilm Life Science, Cambridge, MA, USA).

### Statistical analysis

Statistical analyses were performed with GraphPad Prism v8.1.2 for MacOS (GraphPad Software, San Diego, CA, USA https://www.graphpad.com). Unpaired *t* tests were used to compare the data between two groups and one-way analysis of variance (ANOVA) was used to compare three or more groups. A probability value of less than 0.05 (2-tailed) was considered statistically significant. Multiple testing correction was performed using Bonferroni post-test correction to adjust a significant *p* value.

### Conference presentation

This paper was presented to the 2021 Annual Meeting of the Surgical Research Society and has been published in abstract form—Br J Surg 2021; 108 (S5): znab282.014.

## Results

### Macrophages can be polarized to M1 and M2 macrophages by LPS and IL-4

**Figure 1:**
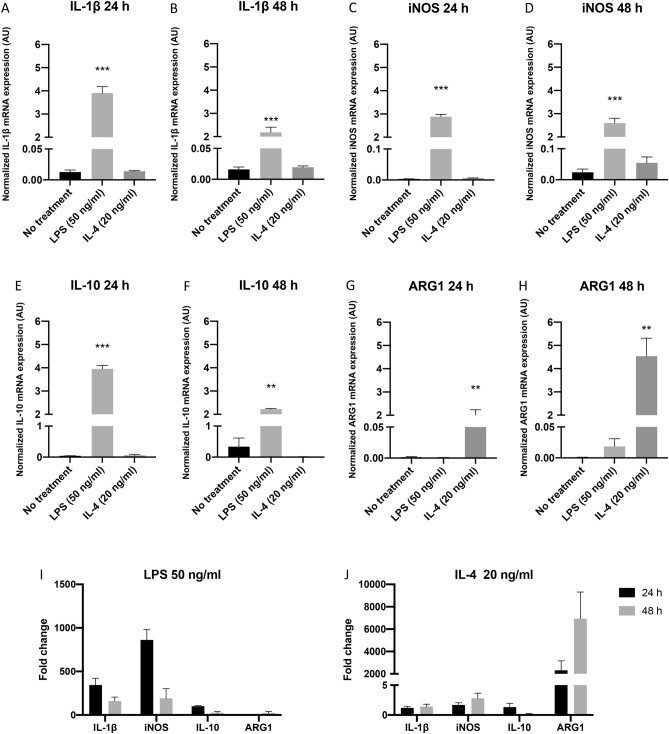
Expression of IL-1β, iNOS, IL-10 and ARG1 mRNA in BMDM cells as markers of their polarization after LPS and IL-4 treatment. (**A–H**) show normalized IL-1β, iNOS, IL-10 and ARG1 mRNA expression levels in BMDMs after treatment with LPS 50 ng/ml or IL-4 20 ng/ml for 24 and 48 h. Data are presented as mean ± SEM of three independent experiments. β-actin was used as an endogenous control for normalization. Statistically significant differences from control (no treatment) were: *p < 0.05, **p < 0.001, ***p < 0.0001 (one-way ANOVA with Bonferroni post-test correction). (**I,J**) show fold changes of IL-1β, iNOS, IL-10 and ARG1 mRNA expression levels in BMDM cells after treatment with LPS 50 ng/ml or IL-4 20 ng/ml for 24 and 48 h compared with untreated cells. *AU* arbitrary units.

To produce M1 and M2 macrophages for the experiments, BMDMs were polarized to form M1 and M2 subtypes by treating with LPS and IL-4, respectively. The expression of IL-1β, iNOS, IL-10 and ARG1 mRNA in BMDM cells was evaluated to demonstrate the polarization efficiency. Expression of IL-1β and iNOS mRNA in BMDMs showed a 344-fold (*p* < 0.0001) and 862-fold (*p* < 0.0001) increase, respectively, in response to LPS treatment at 24 h and then declined considerably at 48 h (Fig. 1A–D,I). In contrast, IL-4 treatment had a minor effect, whether at 24 or 48 h. These results corresponded with M1 polarization of BMDMs by LPS, which had a greater effect at 24 h. IL-10 mRNA expression (Fig. 1E,F) had a similar response as IL-1β and iNOS after LPS and IL-4 treatment, which was inconsistent with its role as an M2 marker. ARG1 mRNA expression, another established M2 macrophage marker, was significantly elevated after IL-4 treatment at 24 h (2318-fold, *p* = 0.0008) and further increased at 48 h (6933-fold, *p* = 0.0008) (Fig. 1G,H,J). The level of ARG1 expression was very low after LPS treatment, validating polarization status.

### M1 macrophage-conditioned media induces PIGR expression in MDA-MB468 cells

To evaluate the effect of polarized macrophages on PIGR expression in breast cancer cells, MCF7 and MDA-MB468 cells were treated with macrophage CM and PIGR mRNA expression of these cells was evaluated by RT-qPCR. M1 CM and M2 CM were prepared from BMDMs after polarization to M1 or M2 macrophages by treatment with LPS for 24 h or IL-4 for 48 h, respectively. CM prepared from untreated BMDMs was M0 CM. To serve as the control, media were prepared using the same process as for M1 CM or M2 CM but without cells in the flasks (No cell CM).

**Figure 2:**
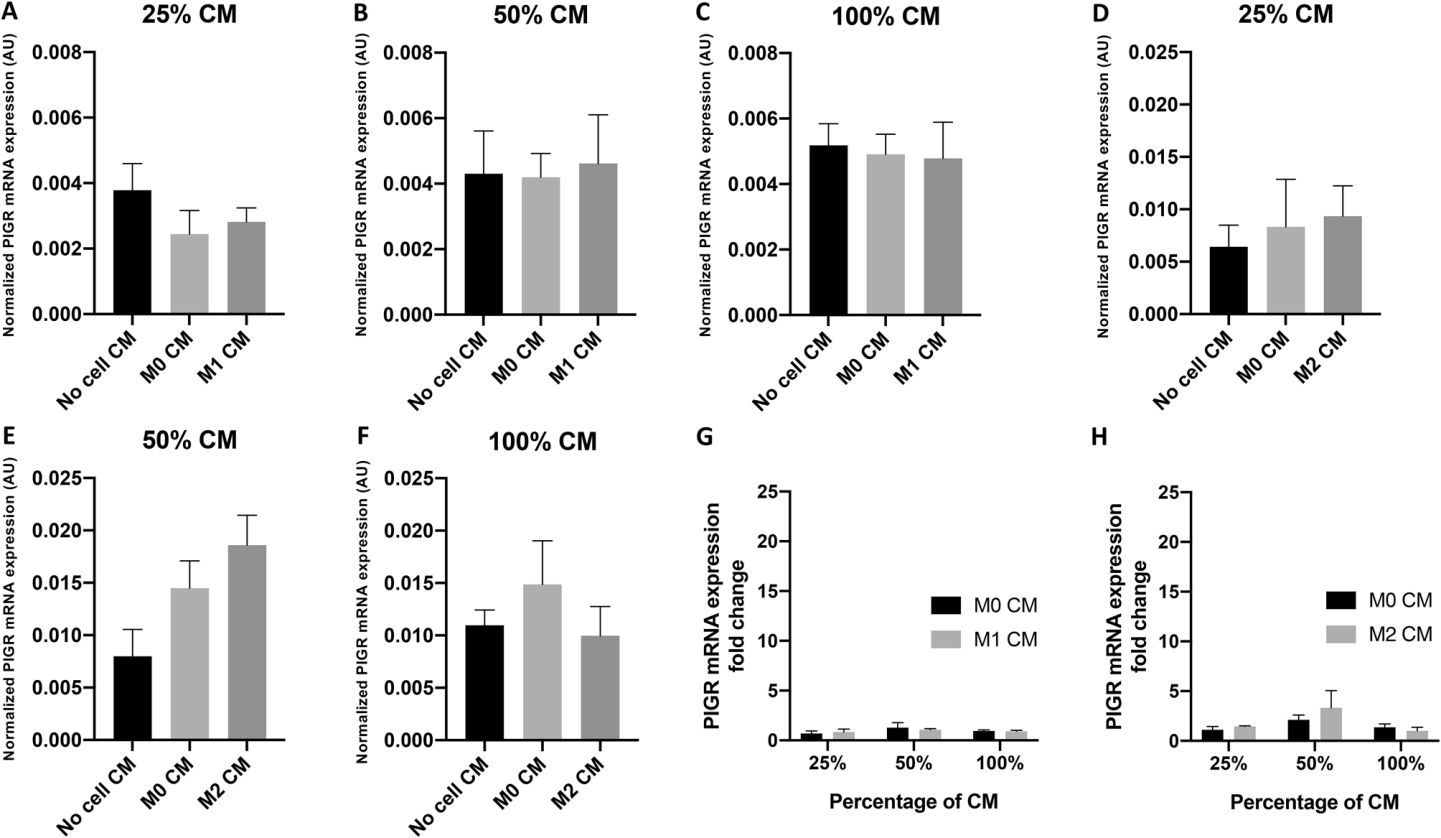
Expression of PIGR mRNA in MCF7 cells after macrophage CM treatment. (**A–F**) show normalized PIGR mRNA expression in MCF7 cells after treatment with 25%, 50% or 100% of no cell, M0 and M1 CM (**A–C**) or M2 CM (**D–F**) for 48 h. Data are presented as mean ± SEM of three independent experiments (no statistically significant difference, one-way ANOVA with Bonferroni post-test correction). GAPDH was used as an endogenous control for normalization. (**G,H**) show fold changes of PIGR mRNA expression in MCF7 cells after treatment with 25, 50 or 100% of M0 and M1 CM (**G**) or M2 CM (**H**) for 48 h from control (no cell CM). Data are presented as mean ± SEM of three independent experiments (no statistically significant difference, multiple *t* test). *AU* arbitrary units.

**Figure 3:**
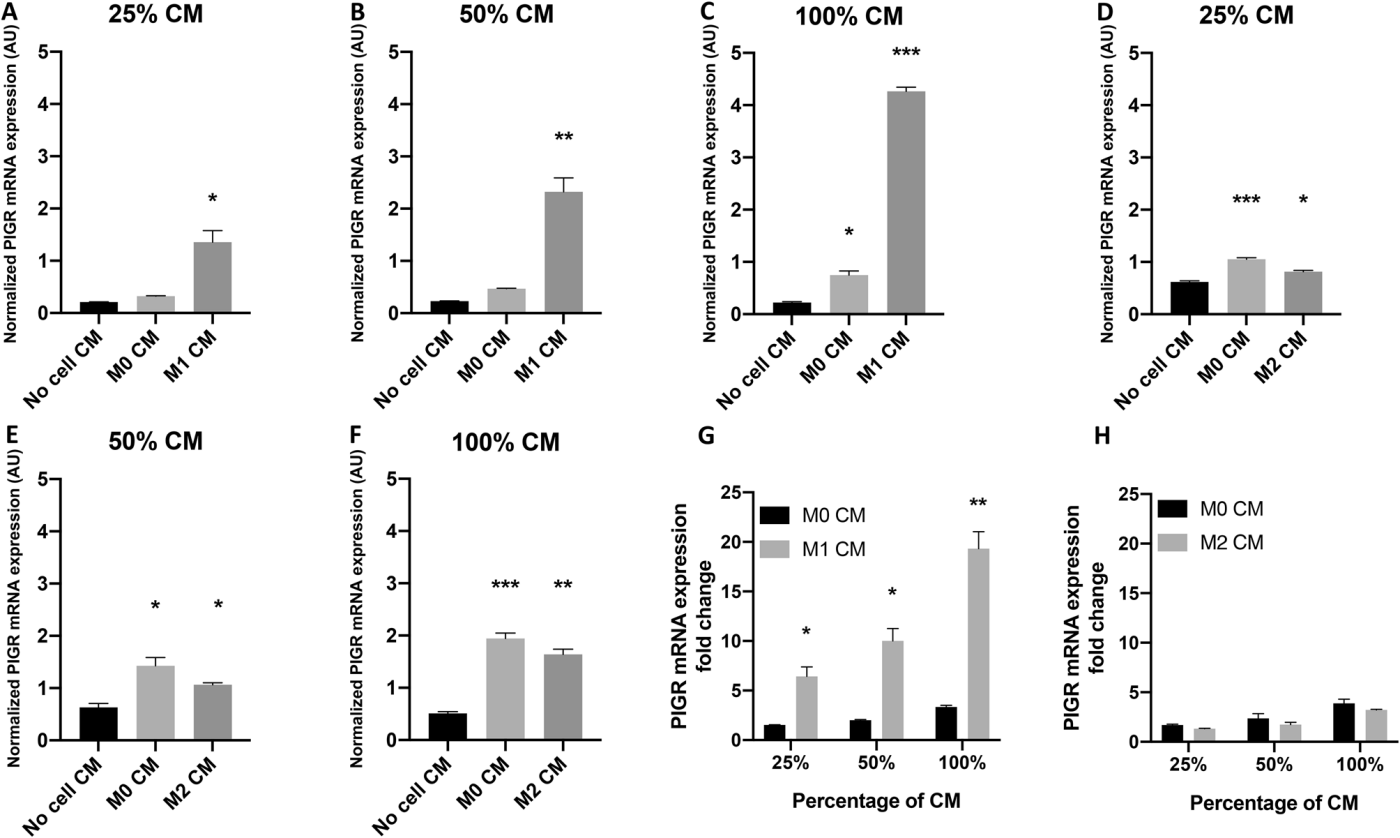
Expression of PIGR mRNA in MDA-MB468 cells after macrophage CM treatment. (**A–F**) show normalized PIGR mRNA expression in MDA-MB468 cells after treatment with 25, 50 or 100% of No cell, M0 and M1 CM (**A–C**) or M2 CM (**D–F**) for 48 h. Data are presented as mean ± SEM of three independent experiments. GAPDH was used as an endogenous control for normalization. Statistically significant differences from control (no cell CM) were: *p < 0.05, **p < 0.001, ***p < 0.0001 (one-way ANOVA with Bonferroni post-test correction). (**G,H**) show fold changes of PIGR mRNA expression in MDA-MB468 cells after treatment with 25, 50 or 100% of M0 and M1 CM (**G**) or M2 CM (**H**) for 48 h from control (no cell CM). Data are presented as mean ± SEM of three independent experiments. Statistically significant differences were: *p < 0.05, **p < 0.001, ***p < 0.0001 (multiple *t* test). *AU* arbitrary units.

Macrophage CM whether M1 or M2 did not significantly change the PIGR mRNA expression level in MCF7 cells compared with M0 CM even when 100% concentration of CM was used (Fig. 2A–H). Nevertheless, the level of PIGR expression in MCF7 cells was very low. PIGR mRNA expression in MDA-MB468 cells was slightly increased after treatment with M0 CM. M1 CM treatment, however, resulted in a significant elevation of PIGR expression in MDA-MB468 cells (25% CM: sixfold, *p* = 0.0075; 50% CM: tenfold, *p* = 0.0031; 100% CM: 19-fold, *p* = 0.0007) (Fig. 3A–C,G). The effects were greater with the higher concentrations of M1 CM treatment. M2 CM treatment significantly increased PIGR mRNA expression in MDA-MB468 but on a smaller scale (Fig. 3D–F,H). Nevertheless, the fold change did not further increase when compared with M0 CM effect.

### IFN-γ but not TNF-α upregulates PIGR expression

**Figure 4:**
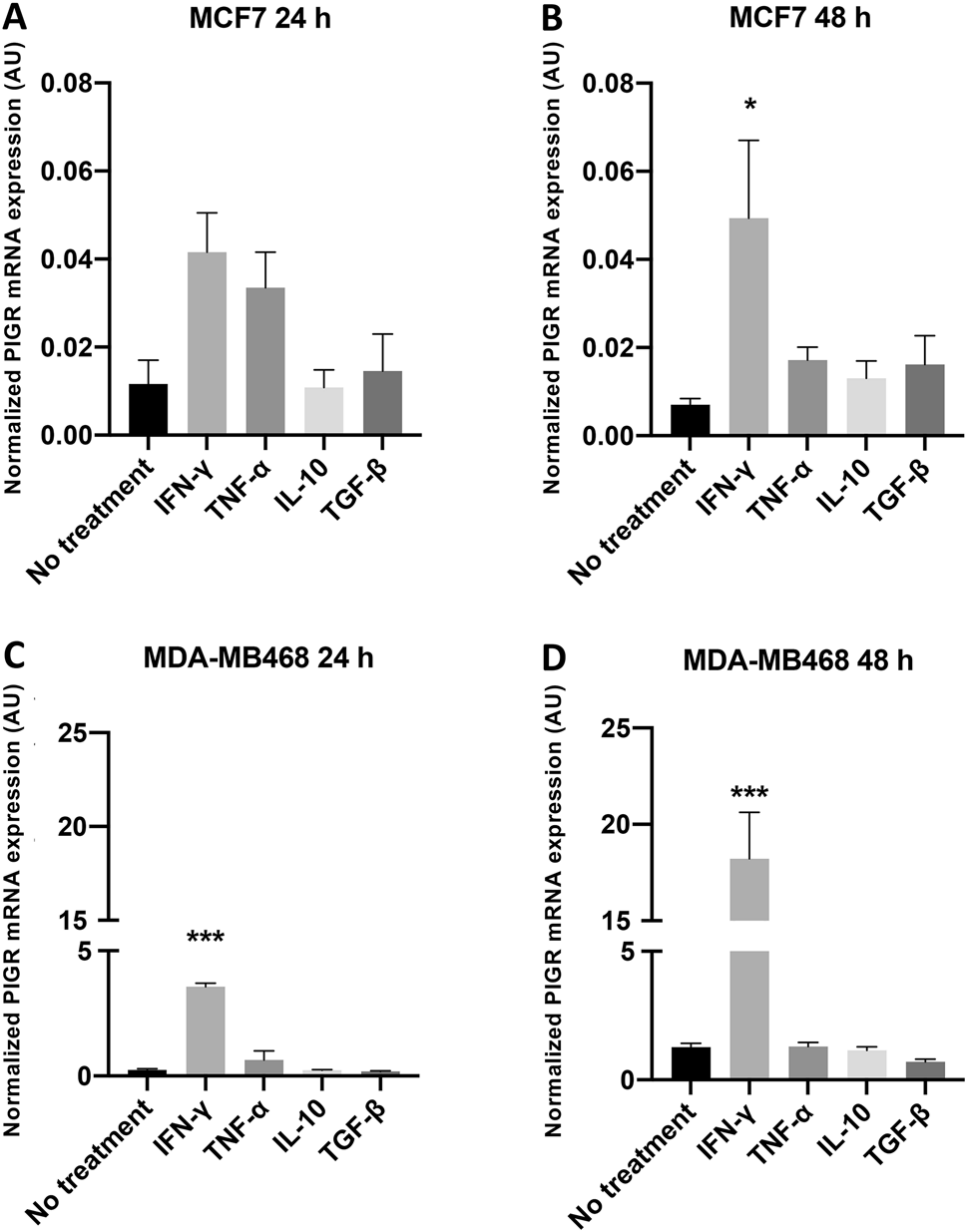
Expression of PIGR mRNA in MCF7 and MDA-MB468 cells after different recombinant cytokine treatment. Normalized PIGR mRNA expression levels in MCF7 (**A,B**) and MDA-MB468 (**C,D**) cells after treatment with IFN-γ 10 ng/ml, TNF-α 10 ng/ml, IL-10 20 ng/ml or TGF-β 5 ng/ml for 24 (**A,C**) and 48 h (**B,D**) are shown. Data are presented as mean ± SEM of three independent experiments. GAPDH was used as an endogenous control for normalization. Statistically significant differences from control (no treatment) were: *p < 0.05, **p < 0.001, ***p < 0.0001 (one-way ANOVA with Bonferroni post-test correction). *AU* arbitrary units.

IFN-γ, TNF-α and TGF-β have been shown to be key cytokines in PIGR regulation in intestinal epithelial cells. However, there were no data in breast cancer cell lines. IL-10 is a pleiotropic cytokine and has been demonstrated to have both proliferative and inhibitory effect on breast tumor cells. The effect of these cytokines on PIGR expression was evaluated. MCF7 and MDA-MB468 cells were treated with recombinant human IFN-γ, TNF-α, IL-10 or TGF-β. The levels of PIGR mRNA expression in the breast cancer cells were evaluated by RT-qPCR. IFN-γ treatment was demonstrated to upregulate PIGR mRNA expression in MCF7 slightly at 24 h and significantly at 48 h (sevenfold, *p* = 0.0259), whereas other cytokines had no significant effect (Fig. 4A,B). The effect of IFN-γ treatment on MDA-MB468 cells was more profound and greater over time. While other cytokines were ineffective, the level of PIGR expression in MDA-MB468 cells showed a 15-fold (*p* < 0.0001) and 26-fold (*p* < 0.0001) increase at 24 and 48 h, respectively, after IFN-γ treatment (Fig. 4C,D).

### IFN-γ mRNA expression was not detected in M1 macrophages

To determine whether IFN-γ was the soluble mediator released by M1 macrophages that was responsible for upregulation of PIGR expression, IFN-γ mRNA expression in BMDMs after polarization to M1 phenotype with LPS for 24 h was evaluated by RT-qPCR. However, IFN-γ expression could not be detected. Shorter time periods of LPS treatment were subsequently assessed. Nevertheless, IFN-γ expression still could not be detected at 1, 2, 4, 8 and 12 h of LPS treatment.

### M1 macrophage-conditioned media induces PIGR expression at least in part, through IL-1β

**Figure 5:**
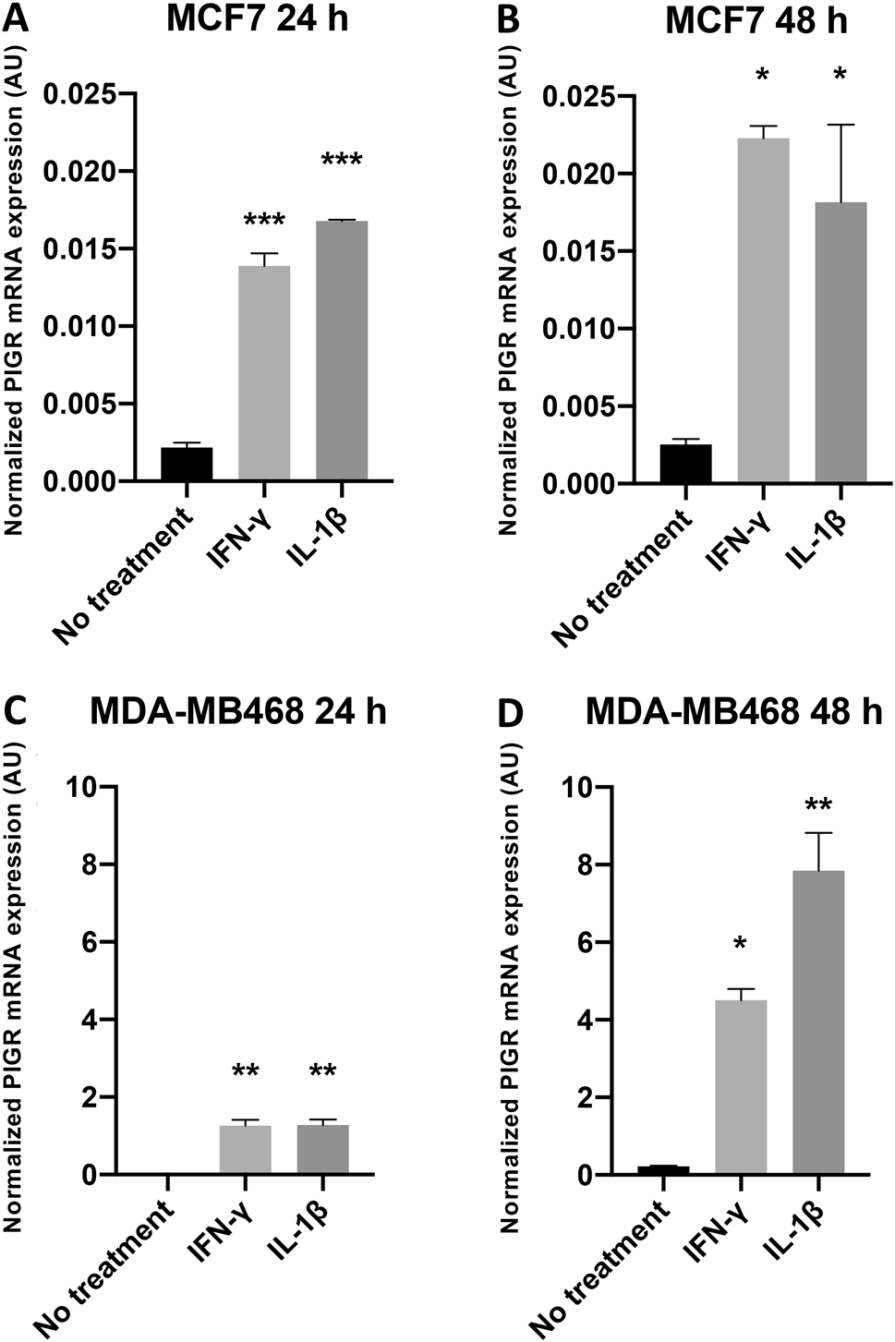
Expression of PIGR mRNA in MCF7 and MDA-MB468 cells after IFN-γ and IL-1β treatment. Normalized PIGR mRNA expression levels in MCF7 (**A,B**) and MDA-MB468 (**C,D**) cell lines after IFN-γ 10 ng/ml and IL-1β 10 ng/ml treatment for 24 (**A,C**) and 48 h (**B,D**) are shown. Data are presented as mean ± SEM of three independent experiments. GAPDH was used as an endogenous control for normalization. Statistically significant differences from control (no treatment) were: *p < 0.05, **p < 0.001, ***p < 0.0001 (one-way ANOVA with Bonferroni post-test correction). *AU*  arbitrary units.

Given the absence of IFN-γ expression in M1 polarized macrophages, we assessed the ability of IL-1β, which was robustly upregulated in M1 polarized cells, to increase PIGR expression in breast cancer cells. IL-1β and IFN-γ significantly increased PIGR expression in MCF7 cells at 24 (IL-1β: eightfold, *p* < 0.0001; IFN-γ: sixfold, *p* < 0.0001) and 48 h (IL-1β: sevenfold, *p* = 0.0185; IFN-γ: ninefold, *p* = 0.0062) but the level of expression was very low (Fig. 5A,B). In MDA-MB468 cells, PIGR mRNA expression also significantly increased at 24 (IL-1β: 44-fold, *p* = 0.0006; IFN-γ: 44-fold, *p* = 0.0006) and 48 h (IL-1β: 36-fold, *p* = 0.0002; IFN-γ: 20-fold, *p* = 0.0042) after IL-1β and IFN-γ treatment (Fig. 5C,D).

**Figure 6:**
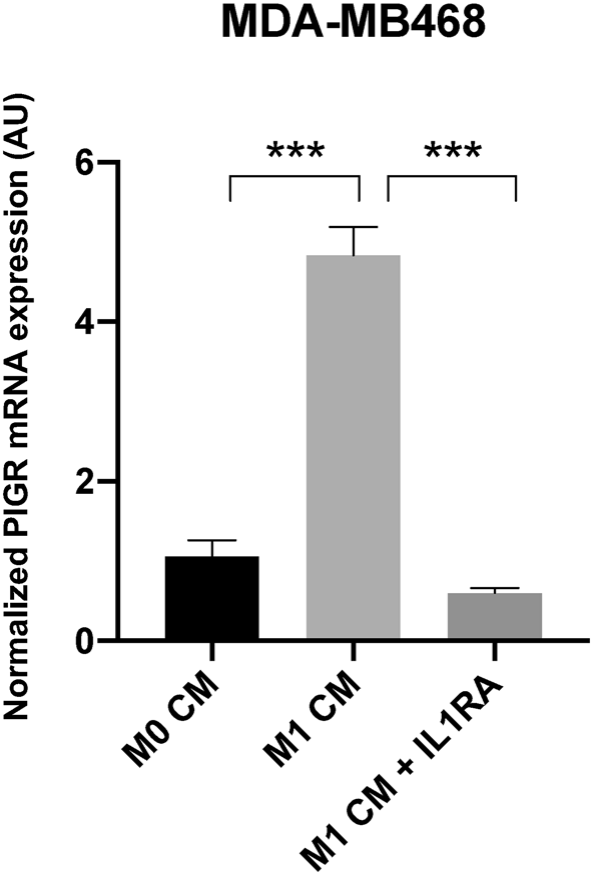
Expression of PIGR mRNA in MDA-MB468 cells after M1 CM with or without IL-1RA treatment. Normalized PIGR mRNA expression in MDA-MB468 cells after treatment with M1 CM with or without IL-1RA for 48 h are shown. Data are presented as mean ± SEM of three independent experiments. GAPDH was used as an endogenous control for normalization. Statistically significant differences from control (No cell CM) were: *p < 0.05, **p < 0.001, ***p < 0.0001 (one-way ANOVA with Bonferroni post-test correction). *AU* arbitrary units.

IL-1 receptor blockade assay using IL-1 receptor antagonist (IL-1RA) was performed to confirm the action of IL-1β secreted from M1 macrophages on the expression of PIGR in breast cancer cells. PIGR mRNA expression in MDA-MB468 cells treated with M1 CM decreased significantly with IL-1RA co-treatment (*p* < 0.0001) (Fig. 6).

### IL-1β increases PIGR expression and the shedding of free SC in a dose-dependent manner in MDA-MB468 cells

**Figure 7:**
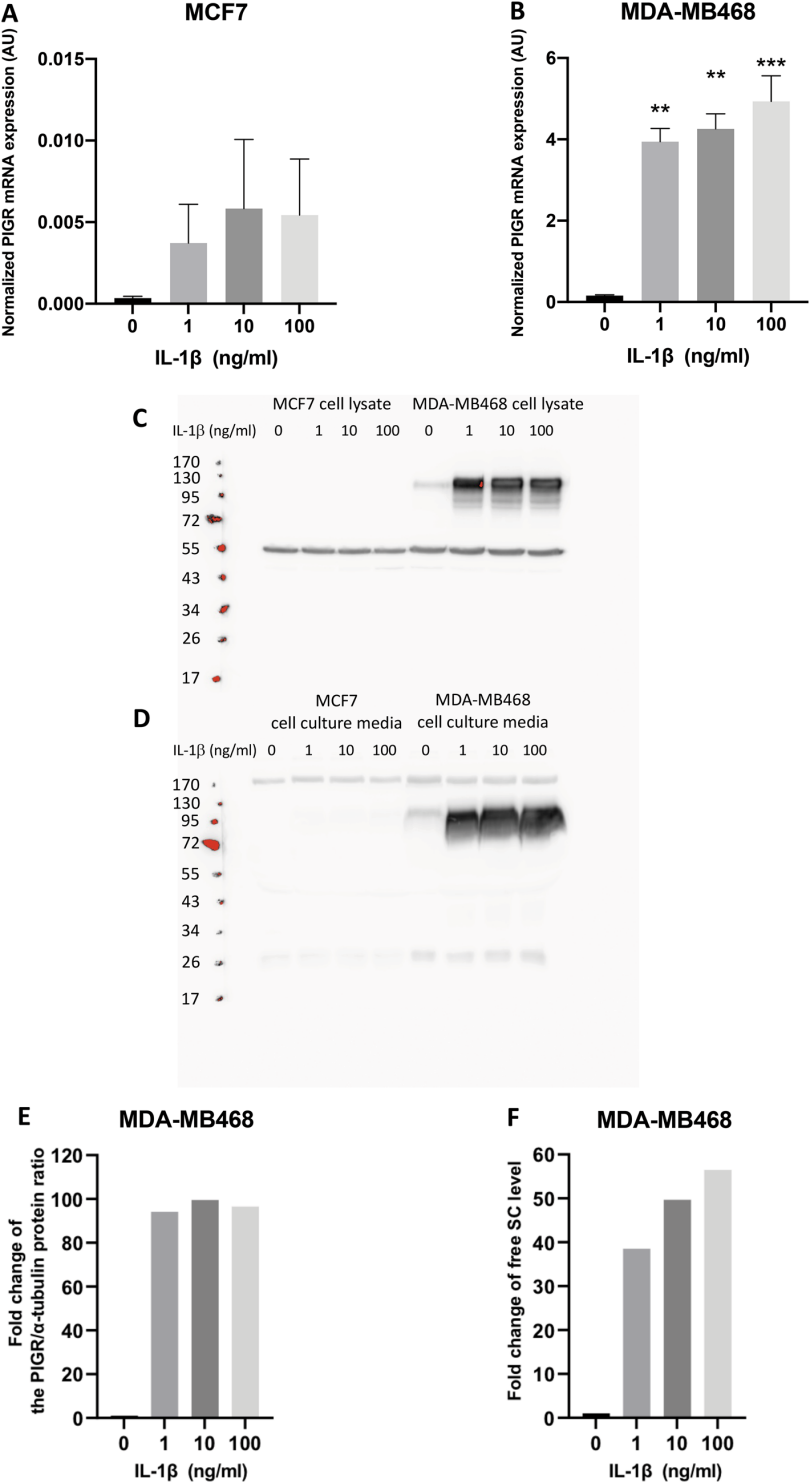
IL-1β dose-dependent effect on PIGR mRNA and protein expression in MCF7 and MDA-MB468 cells and free SC in culture media. Normalized PIGR mRNA expression levels in MCF7 (**A**) and MDA-MB468 (**B**) cells after different concentrations of IL-1β treatment for 48 h are shown. Data are presented as mean ± SEM of three independent experiments. GAPDH was used as an endogenous control for normalization. Statistically significant differences from control (IL-1β 0 ng/ml) were: *p < 0.05, **p < 0.001, ***p < 0.0001 (one-way ANOVA with Bonferroni post-test correction). Representative Western blotting of two independent experiments for PIGR protein expression in MCF7 and MDA-MB468 cell lysates (**C**) and free SC in concentrated cell culture media (**D**) are shown. α-tubulin was used as a loading control for cell lysate. *AU* arbitrary units. PIGR protein expression of MDA-MB468 was quantified and expressed as fold change of the PIGR protein/α-tubulin protein ratio, based on the expression levels observed in untreated cells (**E**). Free SC level was quantified and expressed as fold change based on the level of untreated cells (**F**).

The effect of increasing the concentration of IL-1β on PIGR expression and secretory component secretion in breast cancer cells was further investigated. MCF7 and MDA-MB468 cells were treated with IL-1β 0, 1, 10, 100 ng/ml. PIGR mRNA expression was evaluated by RT-qPCR. PIGR protein expression and free SC secretion were evaluated by Western blotting. MCF7 cells showed a paucity of PIGR mRNA expression even when stimulated with a high concentration of IL-1β (100 ng/ml) (Fig. 7A). IL-1β induced a dose-dependent increase in PIGR mRNA expression in MDA-MB468 cells (Fig. 7B). IL-1β at a low concentration (1 ng/ml) significantly increased PIGR mRNA expression and the expression increased in a dose-dependent manner relative to IL-1β concentration. Intracellular PIGR protein and free SC could not be detected in MCF7 cells (Fig. 7C,D). In contrast, PIGR protein expression in MDA-MB468 cells was dramatically increased with low concentration of IL-1β (1 ng/ml) (Fig. 7E). Free SC was present in MDA-MB468 cell culture media with IL-1β treatment at 1 ng/ml and the levels further increased with 10 and 100 ng/ml treatment, which demonstrated the similar patterns to PIGR mRNA expression in these cells (Fig. 7F).

## Discussion

In the present study, the effect of macrophages and their polarization on PIGR expression in breast cancer cell lines, was investigated. For this purpose, an in vitro model of macrophage polarization was generated and characterized using BMDMs. The CM from polarized macrophages was used to perform the experiments with MCF7 and MDA-MB468 breast cancer cell lines, which are different in their phenotypical characteristics and molecular subtypes. The MCF7 cell line expresses ER and PR but does not express HER2^30^. It is classified as a luminal A subtype, which is less aggressive and non-invasive with low metastatic potential. On the other hand, MDA-MB468 cells lack ER, PR and HER2 expression and are classified as a basal-like subtype, which is an aggressive subtype with poor prognosis^30^.

The results revealed that M1 CM increased PIGR expression in MDA-MB468 cells in a dose-dependent manner, while M2 CM had no effect. These results demonstrated the effect of M1 macrophages on, MDA-MB468 cells, in which the soluble products of M1 macrophages can regulate the expression of PIGR in this basal-like subtype breast cancer cell line. In the host defense mechanism, M1 macrophages release proinflammatory cytokines and have microbicidal capacity. In the TME, M1 macrophages exert tumoricidal activity and elicit tumor destruction^19,20^. Therefore, these findings can supplement and support previous clinical data, which indicated the correlation of high PIGR expression with good prognosis in breast cancer patients that was partly due to the anti-tumoral effect of M1 macrophages in the TME^17^. Furthermore, the expression level of PIGR in breast cancer cells may be a surrogate marker of M1 macrophage infiltration in the TME. However, the level of PIGR mRNA expression in MCF7 cells was very low and was not affected by polarized macrophages whether M1 or M2. Therefore, this may indicate that PIGR is a marker of M1 macrophages only in certain breast cancer subtypes (basal-like subtype).

IFN-γ and IL-1β were shown to significantly increase PIGR mRNA and protein expression in MDA-MB468 cells. The free SC was also documented in cell culture media from MDA-MB468 cells, which clearly demonstrated the functional protein. However, IFN-γ and IL-1β minimally increased PIGR mRNA expression in MCF7 cells which correlated with undetectable protein expression, both intracellularly and in secretion. The responses of breast cancer cell lines to IFN-γ and IL-1β are in keeping with previous reports that IFN-γ and IL-1β increased PIGR expression in human colon carcinoma cell line (HT-29) and lung adenocarcinoma cell line (Calu-3)^31–37^. Nevertheless, a different response was observed for TNF-α, which was demonstrated to increase PIGR expression in the colon carcinoma cell line, while there was no effect on breast cancer cell lines in present study^38^. On the other hand, anti-inflammatory cytokines, IL-10 or TGF-β, did not show any effect on PIGR transcription in both MCF7 and MDA-MB468 cells.

The effective cytokine mRNA expression in M1 macrophages was determined. The result showed that IL-1β but not IFN-γ mRNA was highly expressed in M1 macrophages, suggesting that IL-1β may be an M1 macrophage cytokine involved in PIGR upregulation in breast cancer cells.

## Conclusion

This present study demonstrated a previously unknown interplay between macrophages and breast cancer cells, regarding M1 macrophages enhancing PIGR expression in breast cancer cells through IL-1β. The effect was clearly demonstrated in MDA-MB468 cells which are a basal-like subtype breast cancer. In contrast, MCF7 cells, which are a luminal A subtype, expressed a very low level of PIGR which may not be physiologically relevant. This study also provided evidence that IFN-γ had a similar effect to IL-1β on increasing PIGR expression in breast cancer cells. In the TME, IL-1β is secreted primarily by myeloid cells, not only macrophages but also monocytes and neutrophils^39–41^. IFN-γ is predominantly produced from T lymphocytes and natural killer (NK) cells^42,43^. These imply that the complexity of PIGR regulation in vivo is likely to involve the coordination of various immunomodulatory factors and that the elevation of PIGR expression in breast cancer in vivo may reflect the polarization state of tumor-associated immune cells.

## Supplementary Information

Supplementary Table 1.

## Data Availability

Data will be available on reasonable request from W. Asanprakit wichitraa@hotmail.com.

## Abbreviations

ANOVA: Analysis of variance
ARG1: Arginase 1
AU: Arbitrary units
BMDMs: Bone marrow derived macrophages
CM: Conditioned medium
DMEM: Dulbecco’s modified Eagle medium
EMEM: Eagle’s minimum essential medium
FBS: Fetal bovine serum
HRP: Horseradish peroxidase
Ig: Immunoglobulin
IFN: Interferon
IL: Interleukin
IL-1RA: IL-1: Receptor antagonist
iNOS: Inducible nitric oxide synthase
LPS: Lipopolysaccharide
mRNA: Messenger ribose nucleic acid
MWCO: Molecular weight cut off
NK: Natural killer
PES: Polyethersulfone
PIGR: Polymeric immunoglobulin receptor
pIg: Polymeric immunoglobulin
RIPA: Radioimmunoprecipitation assay
RPMI: Roswell Park Memorial Institute medium
RT-qPCR: Real-time quantitative polymerase chain reaction
SC: Secretory component
SIgA: Secretory IgA
SOCS: Suppressor of cytokine signalling
TAMs: Tumor associated macrophages
TBST: Tris-buffered saline with Tween
TGF-β: Transforming growth factor beta
TME: Tumor microenvironment
