# Supplementary material for: M1 macrophages evoke an increase in polymeric immunoglobulin receptor (PIGR) expression in MDA-MB468 breast cancer cells through secretion of interleukin-1β
Source: Sci Rep. 2022 Oct 7;12:16842. doi: 10.1038/s41598-022-20811-6 (PMC9546936; doi:10.1038/s41598-022-20811-6)
Supplement: Supplementary file 1 — Supplementary Table 1. [file 41598_2022_20811_MOESM1_ESM.docx]

**Supplementary Table 1: Primers and probes sequences for real-time qPCR**

| **Gene** | **Sequence (5’-3’)** |
| --- | --- |
| *hu PIGR* | Forward primer: CAAGATTATCGAAGGAGAACCAAAC  Reverse primer: CCCGTGTTATTCCACTTGCA  Probe: CAAGGTCCCCTGTCACTTTCCATGCA |
| *hu GAPDH* | Forward primer: CAACAGCCTCAAGATCATCAGC  Reverse primer: TGGCATGGACTGTGGTCATGAG  Probe: CCTGGCCAAGGTCATCCATGACAA |
| *ms IL-1β* | Forward primer: TGACAGTGATGAGAATGACCTGTTC  Reverse primer: GGACAGCCCAGGTCAAAGG  Probe: ACCCCAAAAGATGAAGGGCTGCTTCC |
| *ms iNOS* | Forward primer: AGCGAGGAGCAGGTGGAA  Reverse primer: GGAAAAGACTGCACCGAAGATATC  Probe: CCAGCTCAAGAGCCAGAAACGTTATCATG |
| *ms IL-10* | Forward primer: TTTGAATTCCCTGGGTGAGAA  Reverse primer: CTCCACTGCCTTGCTCTTATTTTC  Probe: AGGCGCTGTCATCGATTTCTCCCC |
| *ms ARG1* | Forward primer: GCAGAGGTCCAGAAGAATGGAA  Reverse primer: GCATCCACCCAAATGACACA  Probe: ACCACAGTCTGGCAGTTGGAAGCATCTC |
| *ms IFN-γ* | Forward primer: ACAATGAACGCTACACACTGCAT  Reverse primer: TGGCAGTAACAGCCAGAAACA  Probe: TTGGCTTTGCAGCTCTTCCTCATGG |
| *ms β-actin* | Forward primer: GCTTCTTTGCAGCTCCTTCGT  Reverse primer: GCGCAGCGATATCGTCATC  Probe: CACCCGCCACCAGTTCGCCAT |
